# Spontaneous Neonatal Gastric Perforation: A Case Report

**DOI:** 10.7759/cureus.87877

**Published:** 2025-07-14

**Authors:** Abir Azirar, Mohammed Ech-Chebab, Anass Ayyad, Sahar Messaoudi, Houssain Benhaddou, Rim Amrani

**Affiliations:** 1 Department of Pediatrics, Faculty of Medicine and Pharmacy, Mohammed VI University Hospital, Mohammed First University of Oujda, Oujda, MAR; 2 Department of Neonatology and Neonatal Intensive Care, Mohammed VI University Hospital, Oujda, MAR; 3 Department of Neonatology and Neonatal Resuscitation, Faculty of Medicine and Pharmacy, Mohammed First University of Oujda, Oujda, MAR; 4 Department of Neonatology, Mother and Child Laboratory, Faculty of Medicine and Pharmacy, Mohammed First University of Oujda, Oujda, MAR; 5 Department of Pediatric Surgery, Mohammed VI University Hospital, Oujda, MAR; 6 Faculty of Medicine and Pharmacy of Oujda, Mother and Child Health Laboratory, Oujda, MAR

**Keywords:** gastric perforation, neonate, pneumoperitoneum, prematurity, spontaneous, surgical emergency

## Abstract

Spontaneous gastric perforation (SGP) is a rare but life-threatening surgical emergency in neonates, particularly among preterm infants. We report the case of a male neonate born at 33 weeks of gestation via spontaneous vaginal delivery, with a birth weight of 1,780 grams. The initial postnatal adaptation was satisfactory. However, on the second day of life, the infant developed sudden and severe abdominal distension associated with bilious vomiting and signs of respiratory distress.

A plain abdominal radiograph revealed a massive pneumoperitoneum, highly suggestive of gastrointestinal perforation. The neonate underwent emergency exploratory laparotomy, which revealed a 2 cm linear perforation on the anterior wall of the stomach. The perforation was repaired primarily without the need for gastric resection.

Despite early surgical intervention and aggressive postoperative management in a neonatal intensive care unit, the infant’s condition deteriorated rapidly. He developed signs of sepsis, refractory shock, and multiorgan failure, and succumbed within 48 hours postoperatively.

This case underscores the fulminant course and high mortality rate associated with SGP, especially in preterm neonates. It highlights the critical importance of maintaining a high index of suspicion in the presence of acute abdominal symptoms and radiologic findings, as early diagnosis and prompt surgical intervention are essential for improving outcomes in these vulnerable patients.

## Introduction

Neonatal gastric perforation (NGP) is an uncommon but devastating surgical emergency that typically presents within the first days of life. It is characterized by a sudden full-thickness defect in the gastric wall, often leading to massive pneumoperitoneum and rapid clinical deterioration [1]. Although rare, this condition carries a high risk of morbidity and mortality, especially in premature infants, despite advances in neonatal resuscitation and intensive care [1].

The exact pathophysiology of spontaneous gastric perforation (SGP) remains poorly understood and continues to be the subject of clinical debate [2]. Several hypotheses have been proposed, including congenital defects in the muscular layer of the gastric wall, ischemic injury due to compromised perfusion, mucosal damage related to stress or infection, and increased intragastric pressure secondary to noninvasive ventilation or excessive air swallowing [2]. However, in many cases, no clear etiology can be established.

Early clinical manifestations are often nonspecific and may include sudden abdominal distension, feeding intolerance, respiratory distress, or signs of shock. Because of the nonspecific presentation and the rapid progression of the disease, the diagnosis can be delayed, leading to poor outcomes [2,3]. Radiographic identification of pneumoperitoneum often guides the diagnosis, and emergency laparotomy remains the mainstay of treatment.

Given the rarity and severity of this condition, as well as the importance of rapid recognition and intervention, case reports continue to play a crucial role in raising clinical awareness. We report here a fatal case of SGP in a premature neonate, aiming to underline the key clinical features, diagnostic challenges, and management strategies. We also include a concise literature review to provide context and highlight comparable outcomes.

## Case presentation

A male infant was born prematurely at 33 weeks of gestation via vaginal delivery after an uneventful pregnancy monitored closely for maternal health. The infant’s birth weight was 1,780 grams, and Apgar scores were satisfactory at birth. There was no evidence of maternal infection or complications during labor. Oral enteral feeding was initiated shortly after birth.

At 48 hours of life, the neonate developed acute abdominal distension and bilious vomiting. These alarming symptoms prompted urgent transfer to our neonatal intensive care unit. On arrival, the infant was alert but demonstrated signs of respiratory distress with increased work of breathing and mild cyanosis of the extremities. The respiratory rate was elevated at 48 breaths per minute; the Silverman score was 1/10, indicating mild respiratory distress. Oxygen saturation was 90% on room air. Cardiovascular examination revealed tachycardia at 155 beats per minute and a capillary refill time under three seconds, consistent with hemodynamic stability.

Abdominal examination showed significant distension with diffuse tympany and absent bowel sounds, indicating paralytic ileus or peritoneal irritation. No palpable masses or tenderness were noted. The remainder of the systemic examination was unremarkable.

**Figure 1:**
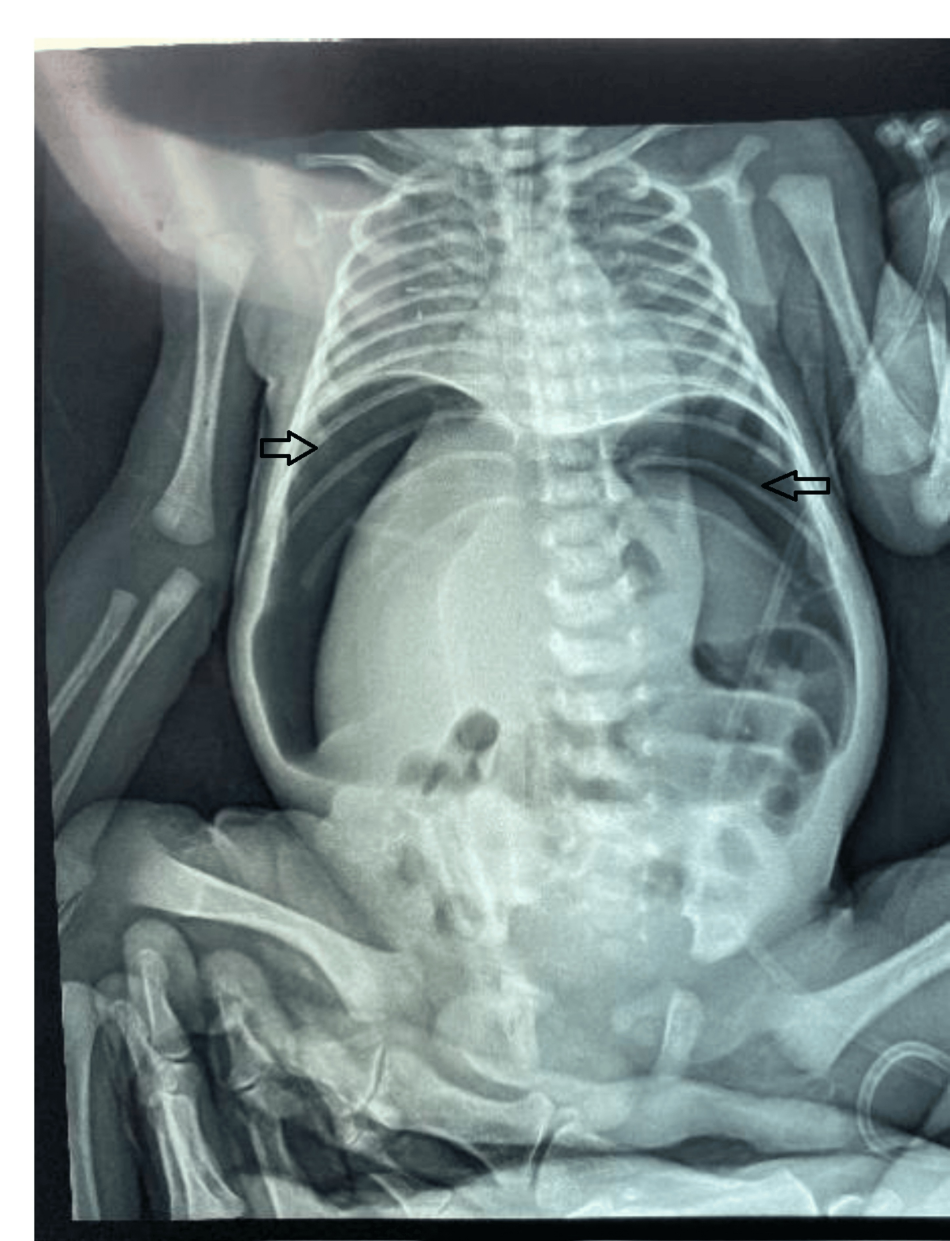
Plain abdominal X-ray demonstrating massive pneumoperitoneum. Note the presence of free air under the diaphragm (indicated by arrows) outlining the abdominal viscera, which is highly suggestive of gastrointestinal perforation

An urgent plain abdominal radiograph demonstrated massive pneumoperitoneum characterized by free air under the diaphragm and outlining the abdominal viscera (Figure 1), strongly suggestive of a perforated hollow viscus. Given the clinical and radiological findings, immediate surgical exploration was indicated.

Preoperative management included gastric decompression via nasogastric tube placement and supplemental oxygen via nasal cannula to support respiratory function. Laboratory investigations, including complete blood count, serum electrolytes, and inflammatory markers, were within normal ranges, suggesting the absence of overt systemic infection at admission.

**Figure 2:**
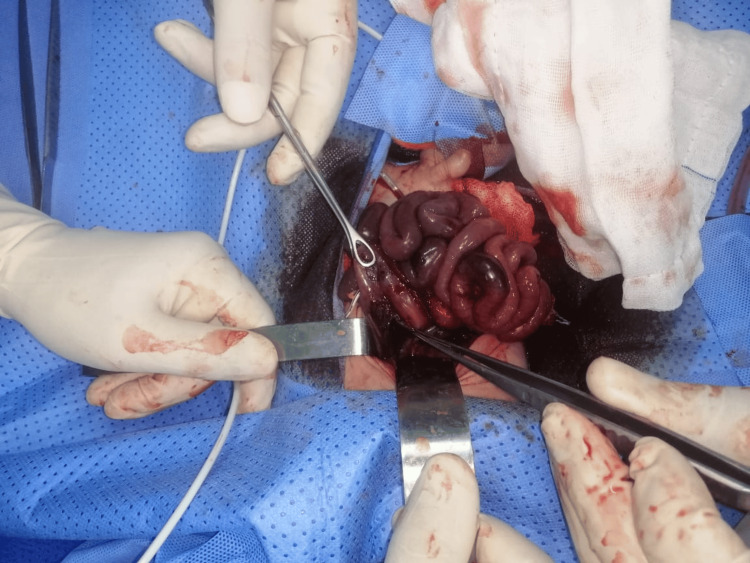
Intraoperative image showing a linear perforation approximately 2 cm in length on the anterior wall of the stomach. The surrounding gastric tissue appears congested but viable. Primary surgical repair was performed at this site

The patient underwent emergency laparotomy, which revealed a linear perforation approximately 2 cm in length located on the anterior wall of the gastric body (Figure 2). The perforation edges appeared viable with no evidence of necrosis. Surgical repair consisted of primary closure of the perforation with interrupted absorbable sutures and thorough peritoneal lavage.

Despite prompt surgical intervention and intensive postoperative care, the infant’s condition deteriorated rapidly due to sepsis and multiorgan failure, resulting in death within 48 hours postsurgery.

## Discussion

SGP in neonates is a rare but life-threatening condition that typically occurs within the first week of life, accounting for approximately 10 to 16% of all neonatal gastrointestinal perforations [2]. Since its first description by Siebold in 1825, fewer than 400 cases have been reported worldwide, highlighting its rarity and the diagnostic challenges it presents [3]. The term “spontaneous” distinguishes this condition from gastric perforations resulting from obstructive lesions, trauma, or iatrogenic causes [4]. Clinically, SGP most commonly manifests between two and seven days after birth, as was the case in our patient, who developed symptoms on the third day of life [5].

Epidemiological data indicate a higher incidence of SGP in male neonates and those of African descent, though cases have been documented across various populations globally. Prematurity is a significant risk factor and is associated with poorer outcomes, likely due to the immaturity of gastric tissues and their increased susceptibility to ischemic injury and stress [1]. Other identified risk factors include low birth weight, premature rupture of membranes, maternal complications such as preeclampsia and diabetes, breech presentation, placenta previa, amniotic infection, and the mode of delivery, with cesarean sections or complicated vaginal deliveries potentially increasing vulnerability [6,7]. In our case, the newborn was male and born prematurely, which is consistent with the risk factors described in the literature.

The pathogenesis of SGP remains controversial and incompletely understood. Several hypotheses have been proposed. One theory suggests a congenital absence or deficiency of the gastric muscularis layer, resulting in a structurally weak gastric wall prone to rupture [7]. However, histological examinations often reveal retracted muscle fibers around the perforation site, which might be a consequence of the rupture rather than its cause [7]. Another possible mechanism involves ischemic injury to the gastric wall caused by vascular insufficiency or systemic hypotension, especially in premature infants who are more prone to hemodynamic instability [8]. Additionally, stress-related mucosal injury, possibly mediated by neurogenic factors, may lead to ulceration and subsequent perforation. A combination of these factors likely contributes to the development of SGP in vulnerable neonates [7].

Clinically, NGP presents abruptly, with symptoms including sudden abdominal distension, vomiting often bilious, and rapid onset of respiratory distress due to diaphragmatic irritation from free intraperitoneal air. The condition rapidly progresses and, if left untreated, can lead to sepsis, multiorgan failure, and death [9]. Radiological examination with a plain abdominal X-ray remains the cornerstone of diagnosis, with pneumoperitoneum appearing as a hallmark feature. While ultrasound and other imaging modalities may provide additional information, they are less definitive in acute settings [9,10].

Management requires urgent surgical intervention. Laparotomy with primary repair of the perforation is the standard approach. In some cases, preoperative abdominal decompression by needle aspiration has been used to relieve respiratory distress caused by massive pneumoperitoneum [7,9,10]. Despite advances in neonatal intensive care and surgical techniques, mortality remains high, especially when diagnosis and treatment are delayed, or when the infant is premature or septic at presentation [9]. Our case underscores these challenges, as despite timely surgical repair, the neonate succumbed to postoperative complications.

## Conclusions

Spontaneous neonatal gastric perforation (SNGP) remains an exceptionally rare but potentially fatal surgical emergency, primarily affecting premature infants. Despite advances in neonatal care and surgical techniques, this condition continues to be associated with a high mortality rate due to its sudden onset, nonspecific early signs, and rapid progression to septic shock and multiorgan failure. This case highlights the dramatic clinical course SNGP can take, even with prompt diagnosis and timely surgical intervention. The infant’s condition deteriorated rapidly despite rigorous management, underscoring the severity of this pathology. This emphasizes the need for clinicians, particularly neonatologists, pediatric surgeons, and emergency teams, to maintain a high level of suspicion in any newborn presenting with sudden abdominal distension, vomiting, and respiratory distress, especially during the early postnatal period or in the context of prematurity. Early and appropriate radiological evaluation, typically revealing pneumoperitoneum, remains the cornerstone of diagnosis. However, given the diagnostic challenge posed by the nonspecific nature of the initial symptoms, a low threshold for imaging should be adopted in symptomatic neonates. Once the diagnosis is established, urgent surgical exploration is crucial to prevent rapid deterioration and fatal outcomes. In summary, this case underscores the aggressive nature of SGP in neonates and the vital importance of early diagnosis and surgical management. It also highlights the need for continued research into the etiology and prevention of this rare but catastrophic condition.

## Figures and Tables

**Figure 3:**
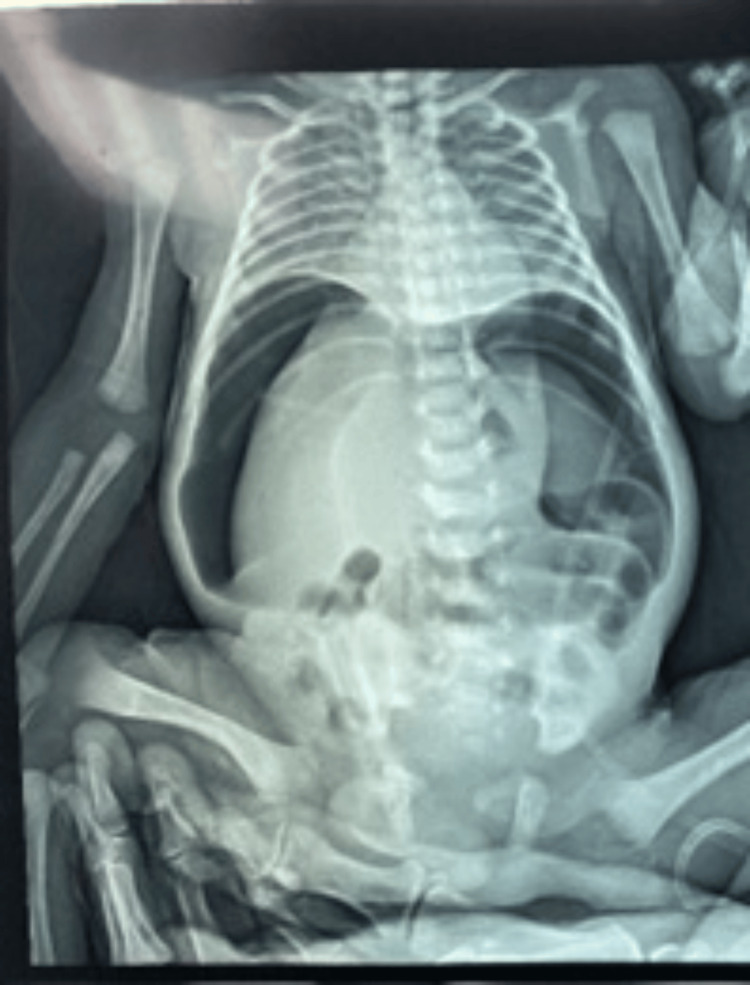
The plain abdominal X-ray showed significant pneumoperitoneum

**Figure 4:**
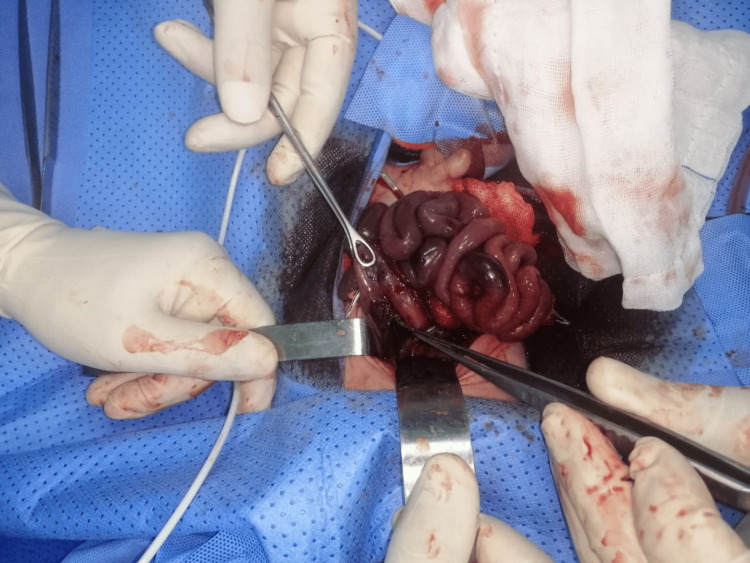
Gastric perforation during laparotomy
